# Cryo‐Induced Hypoalgesia: The Effects of an Acute Cryochamber Exposure on Pain Perception—A Randomised Controlled Cross‐Over Trial

**DOI:** 10.1002/ejp.70017

**Published:** 2025-04-04

**Authors:** Fabian Tomschi, Alexander Schmidt, Thomas Cegla, Thomas Hilberg

**Affiliations:** ^1^ Department of Sports Medicine University of Wuppertal Wuppertal Germany; ^2^ Department of Pain Medicine Helios University Hospital Wuppertal Wuppertal Germany

**Keywords:** cryochamber, pain sensitivity, pressure pain thresholds, whole‐body cryotherapy

## Abstract

**Background:**

Whole‐body cryotherapy (WBC) is a treatment that involves exposing the entire body to extremely cold temperatures and is used in therapeutic and sports scientific settings. Here, cryotherapy is often used to alleviate pain, but its underlying pain physiological effects are under‐researched. This study aims to explore whether a 3‐min cryochamber application results in cryo‐induced hypoalgesia.

**Methods:**

24 healthy male participants successfully conducted this randomised controlled crossover study consisting of a 3‐min WBC (cryochamber at −87°C) and a 3‐min control (ambient temperature) session. Pressure pain thresholds (PPT [Newton/cm^2^]) were measured at the rectus femoris, knee joint, deltoid muscle and sternum pre and post0, post5, post15 and post30.

**Results:**

Results revealed a significant ‘condition’ × ‘time’ interaction (*p* < 0.001, *η* ^2^_partial_ = 0.280) for PPT_Total_ (pooled for one average value) with hypoalgesia observed after WBC (*p* < 0.001; pre: 77.0 ± 17.2, post0: 89.6 ± 18.6, post5: 83.6 ± 19.4, post15: 83.1 ± 18.2, post30: 80.8 ± 17.7) and no change following the control (*p* = 0.873; pre: 75.1 ± 18.8, post0: 75.3 ± 19.4, post5: 74.6 ± 19.2, post15: 75.7 ± 19.3, post30: 75.3 ± 19.1). The same pattern was observed for individual landmarks. Between‐group differences were consistently observed, with higher values following the WBC. No significant ‘time’ × ‘intervention’ × ‘landmark’ interaction effect (*p* = 0.439) was found.

**Conclusions:**

This study demonstrates that a three‐minute cryochamber exposure induces robust hypoalgesia in healthy participants, as indicated by increased PPT, lasting up to 30 min but gradually declining over time.

**Significance Statement:**

Whole‐body cryotherapy significantly induces short‐term hypoalgesia, with increased mechanical pain thresholds lasting up to 30 minutes. Compared to ambient temperature, cryotherapy provided greater pain relief across multiple body landmarks, with 82.6% of participants experiencing hypoalgesia. These findings highlight the potential of cryotherapy for pain management and support further research on its long‐term efficacy and clinical applications.

## 1 Introduction

Cold applications have gained increasing interest in research and especially in the sports and clinical fields due to their proposed benefits in pain management and recovery enhancement (Jdidi et al. 2024; Patel et al. 2019). A whole spectrum of different cold modalities exists, ranging from localised applications with ice packs to more systemically effective forms such as, for instance, cold‐water immersion or cryochambers (Bouzigon et al. 2016). These modalities leverage the physiological response of various bodily systems to cryogenic temperatures, which include, among others, vasoconstriction and a reduction in nerve fibre conduction velocity for the alleviation of pain and inflammation (Herrera et al. 2010; Swenson et al. 1996). Afterwards, vasodilatation of the microvasculature leads to an increase in body temperature above baseline levels, a phenomenon that may persist for an extended period of time (Dębiec‐Bąk et al. 2013). Furthermore, an upregulation of parasympathetic activity measured by means of heart rate variability can be observed after termination of whole‐body cryotherapy (Louis et al. 2020). Cold exposures are administered to athletes to positively manipulate regeneration and reduce muscle soreness after training and competition (Bleakley et al. 2012; Versey et al. 2013). From a medical point of view, cryotherapy can be utilised as an acute or long‐term non‐pharmacological treatment option for pain due to its analgesic potential (Kunkle et al. 2021).

One specific type of whole‐body cryotherapy (WBC) involves staying inside a cryochamber. A cryochamber is an enclosed space where individuals are subjected to extremely cold temperatures ranging from −60°C up to −140°C for a brief duration, up to a maximum of usually 3 min (Dębiec‐Bąk et al. 2013; Stanek et al. 2018).

As mentioned before, pain modulation is one crucial and often exploited benefit of acute or repetitive cold exposure. To investigate changes in pain perception across various settings, pressure pain thresholds (PPT) are frequently used, with higher PPT indicating less pain sensitivity. Assessment of PPT offers an experimental and semi‐objective method for evaluating an individual's pain sensitivity (Böing‐Meßing et al. 2022; Tomschi, Schmidt, et al. 2024), providing localised and tissue‐specific responses to experimental and clinical interventions such as cold exposure. The body of evidence on the effects of cryogenic temperatures on pain thresholds is sparse and currently limited to localised application methods (Algafly and George 2007; Curković et al. 1993; Macedo et al. 2015; Vargas E Silva et al. 2019). To the best of the authors' knowledge, it has not been determined empirically whether whole‐body cryotherapy modulates an individual's pain sensitivity.

Based on the above‐mentioned considerations, this study seeks to explore whether a 3‐min cryochamber application of −87°C results in cryo‐induced hypoalgesia. More particularly, the following research questions are stated: (1) Does a cryochamber application result in acutely increased mechanical PPT (i.e., hypoalgesia) compared to a control condition? (2) How long does the potential increase in PPT last? (3) Do tissue‐specific differences in PPT exist?

## 2 Materials and Methods

### 2.1 Study Design and Examination Procedure

**FIGURE 1:**
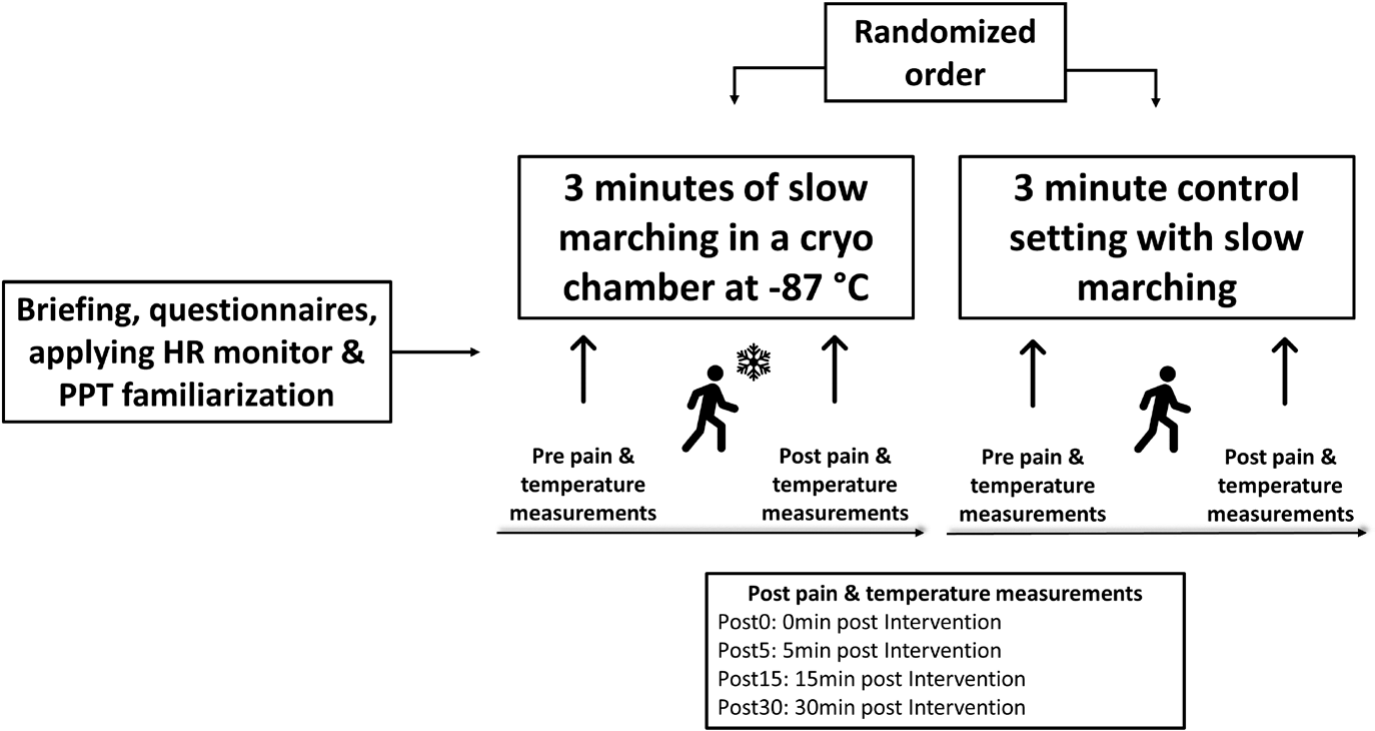
Schematic overview of the study design. HR, heart rate; PPT, pressure pain thresholds.

This study was carried out as a randomised controlled cross‐over trial in which participants conducted one cryochamber and one control session in a randomised order (Figure 1). Simple randomisation was used for the random allocation sequence, and blinded allocation was ensured using a randomisation system by an independent researcher. To avoid any carryover effects, a washout period of at least 7 days was administered.

In the first visit, the contents of the study were explained and participants provided written consent. Then, PPT measurements were performed to familiarise participants with this assessment to avoid any confounding results due to the novelty of the measurement. Following that, participants changed their clothing. For protection and discretion, participants wore their own underpants, a hat, gloves, socks, shoes and a medical face mask. This clothing pattern was standardised for both conditions and across all measurement time points. Further, to prevent excessive cooling of the body, entering the cryochamber was only permitted when the skin surface was completely dry. PPT and local temperature were measured in a seated position prior to the experimental phase (Pre). Participants were then instructed to march slowly for 3 min, either in the cryochamber cooled to a constant temperature of −87°C (cryochamber intervention) or in a quiet room at ambient temperature (control condition). During the experimental phase, heart rate (HR) was monitored. Subsequently, PPT, temperature and HR were assessed immediately post the experimental phase (Post0), after 5 min (Post5), 15 min (Post15) and 30 min (Post30). Reporting of this study was performed according to the CONSORT checklist for randomised controlled crossover trials (Dwan et al. 2019) and no changes to methods after inclusion of the first participant were done.

### 2.2 Ethics

This study was conducted in accordance with the principles of good clinical and ethical practice and was approved by the local ethics committee (Number: SK/AE 230109, date of approval: 27 January 2023). Along the Declaration of Helsinki, participants were informed in detail about the study protocol prior to the examination and were required to give written informed consent.

### 2.3 Participants

Based on a priori power analysis conducted with G*power (Version 3.1.9.4) with a predefined alpha level of 0.05, a power of 0.8, and a moderate effect size of *f* = 0.25 (equals *d* = 0.5), the recruitment of at least 22 participants was required. Given that, to date, no study has assessed PPT applying whole‐body cryotherapy, we chose the aforementioned standardised moderate effect size. To account for potential dropouts of approximately 10%, a total of 25 healthy male individuals were recruited and enrolled in the study. Eligibility for study participation required the submission of written informed consent, being male, and an age of at least 18 years. In addition, participants were excluded if they took pain medication regularly or had taken any pain medication 24 h prior to examination. Participants were also asked to refrain from performing any type of vigorous exercises 48 h prior to any study visit that might induce muscle soreness. Finally, the presence of a disease contraindicated for the cryochamber (e.g., PAD, CHD, bronchial asthma, Raynaud's disease) or being diagnosed with a musculoskeletal disease (e.g., osteoarthritis, rheumatoid arthritis) precluded participation.

### 2.4 Pressure Pain Thresholds

PPT were examined using a digital pressure algometer (Wagner Instruments, Greenwich, Connecticut, USA). Studies have shown excellent inter‐rater reliability for PPT assessment in healthy individuals at different body regions (Jayaseelan et al. 2021; Waller et al. 2015). The precise assessment procedure was similar to the study conducted by Tomschi et al. (2022), with the adjustment that each landmark was assessed twice consecutively. In short, the pressure was constantly increased (10 Newton/s) until the subject verbally indicated that the pressure first became painful. Peak pressure was set at 120 Newton/cm^2^ to avoid the risk of tissue irritation. At each landmark, two measurement series were conducted, and the average value was used for statistical analyses. Higher values indicate a lower level of pain sensitivity.

To ensure standardised assessment of outcomes, the body areas were marked prior to the first measurement series. Landmarks assessed for PPT comprised the rectus femoris muscle (RF) at the centre of the distal third between the patella base and the anterior superior iliac spine (Vaegter et al. 2018) and the lateral area of the acromial part of the deltoid muscle (DM) (Persson et al. 2004) as muscular measurement points. The knee joint (KJ) between the medial condyle of the femur and the medial condyle of the tibia (Tomschi, Ransmann, et al. 2024) and the sternum (ST) two finger widths above the xiphoid process were determined as non‐muscular measurement points (Tomschi et al. 2023). All PPT measurements were performed in the same presented sequence. PPT assessment of each participant was carried out by the same investigator.

### 2.5 Temperature and Heart Rate

The skin surface temperature was measured using an infrared non‐contact thermometer (FIT‐10 Thermometer, PCE, Meschede, Germany) consistently before the assessment of PPT at the exact same landmarks as described above. The probe was placed at the designated landmark, and temperature readings were recorded to the nearest 0.1°C. This specific thermometer was selected because it is portable, inexpensive, easy to use, and fast to use and demonstrated good inter‐rater reliability in a similar model (ICC = 0.80) (Packham et al. 2012).

HR (Polar m400; Polar Electro OY, Kempele, Finland) was monitored in 10 s intervals throughout the experimental phase and at each aforementioned measurement time point.

### 2.6 Statistics

Statistical analyses were conducted using IBM SPSS 29 (Armonk, NY, USA) for Windows with a predefined alpha level of *p* ≤ 0.05. Figures were created using RStudio (version 4.2.3), primarily utilising the package ‘ggplot2’. Data was initially tested for normal distribution employing Shapiro–Wilk tests and by inspection of Q–Q plots. As PPT represents the main outcome variable and distributions failed to elicit normality in some cases, the data were log10‐transformed as recommended by Rolke (Rolke et al. 2006) and were, hence, presented as mean and standard deviation.

Subsequently, to analyse the effect of the cryochamber intervention compared to the control condition on PPT across all measurement time points, a 2 × 5 ANOVA was performed for the mean PPT of all landmarks (PPT_Total_). To check for sphericity, Mauchly's Test was performed, and in case of heterogeneity of variances within groups, Greenhouse–Geisser correction was used. Further, a three‐way ANOVA with the main factors ‘time’ (Pre, Post0, Post5, Post15, Post30), ‘condition’ (Cryochamber, Control) and ‘landmark’ (RF, DM, KJ, ST) was conducted to analyse the distinct effects of the two interventions on PPT over time and to analyse whether the hypoalgesic effects are dependent on the measurement site assessed. In case of significant differences, LSD post hoc tests were applied (Lee and Lee 2018). Effect sizes were calculated as partial eta‐squared (*η* ^2^_partial_) and interpreted as follows: *η* ^2^_partial_ ≥ 0.01 = small effect, *η* ^2^_partial_ ≥ 0.06 = medium effect, *η* ^2^_partial_ ≥ 0.14 = large effect (Cohens 1988).

Lastly, responsiveness to the cryochamber intervention was determined by comparing post measurement data for PPT to Pre‐values relative to the standard error of measurement (SEM). To compute SEM values for each landmark, both Pre‐values for each condition were used, applying the following formula: $\mathrm{SEM}=\text{SDpooled}*\sqrt{1-\mathrm{ICC}}$, with ICC meaning the intraclass correlation coefficient. For this analysis, only participants with baseline PPT values smaller than 120 Newton (upper cut‐off) minus SEM were considered, as only these data allow for the identification of true responders.

## 3 Results

### 3.1 General Results

In the course of this study lasting from March 2023 to November 2023, one drop‐out was recorded. One participant terminated the cryochamber intervention before the 3‐min duration due to discomfort, and data from this participant are not included in any analysis. No further events nor any adverse events or harms were observed. Therefore, a total of 24 healthy males successfully completed the study and were subsequently analysed. Subject characteristics, including data regarding the cryochamber intervention, can be obtained from Table 1. No subject had experience with WBC, nor did any subject report regularly performing cold exposures. Seven participants reported having experience with cold exposures, such as cold showers or cold water immersion.

**TABLE 1:** Subject characteristics (*n* = 24).

Parameter	Healthy individuals
Age (years)	27.8 ± 2.8 (24.0–34.0)
Height (m)	1.82 ± 0.07 (1.57–1.95)
Weight (kg)	78.3 ± 9.3 (48.0–95.5)
BMI (kg/m^2^)	23.7 ± 1.6 (19.5–26.7)
NRS_cryo_	8.9 ± 1.0 (7.0–10.0)
Prior experience to cold exposures	No: 17 (70.8%), Yes: 7 (29.2%)

*Note:* Data presented as mean ± standard deviation (Min–Max).

Abbreviations: BMI, Body‐mass‐index; NRS_cryo_, sensation of cold on a numeric rating scale from 0 (no cold) to 10 (maximum cold sensation imaginable).

**FIGURE 2:**
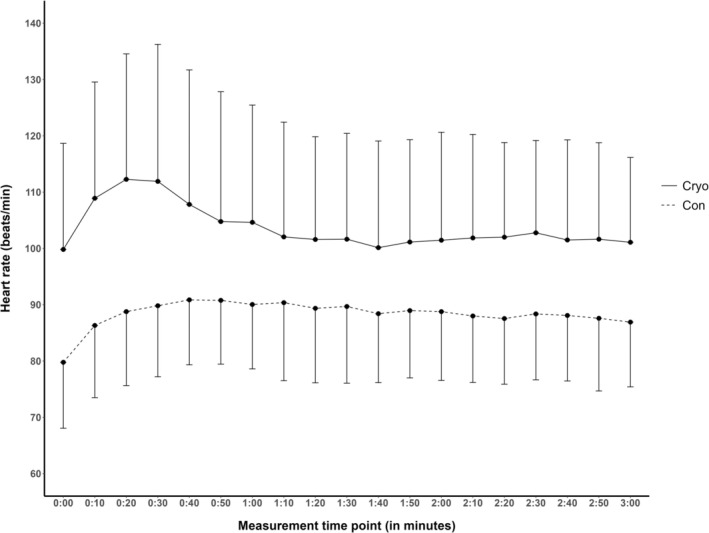
Changes in heart rate of participants (*n* = 24) during the cryochamber intervention (Cryo) and control condition (Con). Data are presented as means with whiskers representing standard deviation.

Descriptive data of HR during the cryochamber intervention and temperature for the designated five measurement time points can be obtained from Figure 2 and Table 2, respectively.

**TABLE 2:** Temperature in °C for muscular and bony landmarks at each measurement time point (*n* = 24).

Landmark	Condition	Pre	Post0	Post5	Post15	Post30
Muscular						
RF	Cryo	31.6 ± 1.2 (29.4–34.1)	20.4 ± 3.7 (11.8–27.6)	27.4 ± 2.1 (23.6–31.3)	29.9 ± 2.7 (19.4–33.4)	30.8 ± 1.5 (28.3–34.0)
	Con	31.1 ± 1.1 (29.5–33.1)	30.8 ± 1.1 (29.0–32.9)	31.3 ± 1.2 (28.8–33.2)	31.5 ± 1.1 (29.3–33.7)	31.7 ± 1.4 (29.2–34.2)
DM	Cryo	34.1 ± 0.7 (32.7–35.5)	25.4 ± 2.2 (21.2–29.6)	29.9 ± 3.3 (18.7–34.0)	31.9 ± 1.6 (29.1–35.6)	32.4 ± 1.4 (29.1–34.3)
	Con	33.6 ± 0.7 (32.1–34.7)	33.7 ± 1.0 (31.2–35.3)	33.8 ± 0.8 (32.5–35.7)	33.9 ± 1.0 (32.4–36.0)	33.6 ± 1.0 (32.0–36.0)
Bony						
KJ	Cryo	30.2 ± 1.3 (27.8–33.0)	22.4 ± 2.3 (16.6–26.0)	26.4 ± 2.5 (17.4–30.4)	27.9 ± 1.6 (26.0–31.7)	28.4 ± 1.5 (26.2–30.9)
	Con	30.5 ± 1.3 (27.8–33.2)	30.2 ± 1.5 (28.1–33.4)	30.1 ± 1.4 (28.3–33.6)	30.0 ± 1.4 (27.1–32.6)	29.7 ± 1.5 (27.5–33.2)
ST	Cryo	34.0 ± 1.1 (29.8–35.6)	27.9 ± 2.2 (21.7–30.8)	30.8 ± 1.4 (26.1–33.0)	32.4 ± 1.3 (29.1–35.0)	33.2 ± 1.0 (31.6–35.2)
	Con	33.6 ± 1.2 (30.0–35.0)	33.7 ± 1.2 (29.8–35.5)	33.5 ± 1.4 (29.9–35.3)	33.9 ± 0.9 (31.3–35.0)	34.1 ± 0.8 (32.1–35.3)

*Note:* Data presented as mean ± standard deviation (Min–Max).

Abbreviations: DM, deltoid muscle; KJ, knee joint; RF, rectus femoris muscle; ST, sternum.

### 3.2 Changes in Pressure Pain Thresholds

**FIGURE 3:**
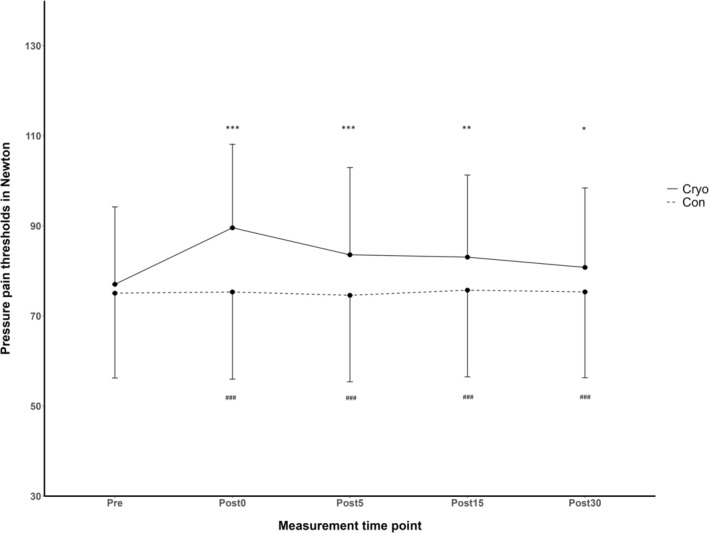
Changes in total pressure pain thresholds in participants (*n* = 24) before and after the cryochamber intervention (Cryo) and control condition (Con). *p ≤ 0.05, **p ≤ 0.01, ***p ≤ 0.001 for within‐group differences compared to Pre. ^###^ *p* ≤ 0.001 for between‐group differences at the respective time point. Data are presented as mean with whiskers representing standard deviation.

For PPT_Total_, the two‐way ANOVA demonstrated a significant interaction effect (*p* < 0.001, *η* ^2^_partial_ = 0.280). Respective post hoc pairwise comparisons revealed for the cryo chamber intervention that PPT_Total_ values significantly decreased from Pre to Post0 (*p* < 0.001), from Pre to Post5 (*p* = 0.001), from Pre to Post15 (*p* = 0.003), and from Pre to Post30 (*p* = 0.035). In the control condition, no significant changes were observed between Pre and any post‐intervention time points (all *p* > 0.582). Comparisons between the cryo chamber and control conditions at each time point showed no differences for PPT_Total_ at Post0 (*p* = 0.290). Significant differences were observed at Post0 (*p* < 0.001), Post5 (*p* < 0.001), Post15 (*p* = 0.001) and Post30 (*p* = 0.001) (Figure 3).

**FIGURE 4:**
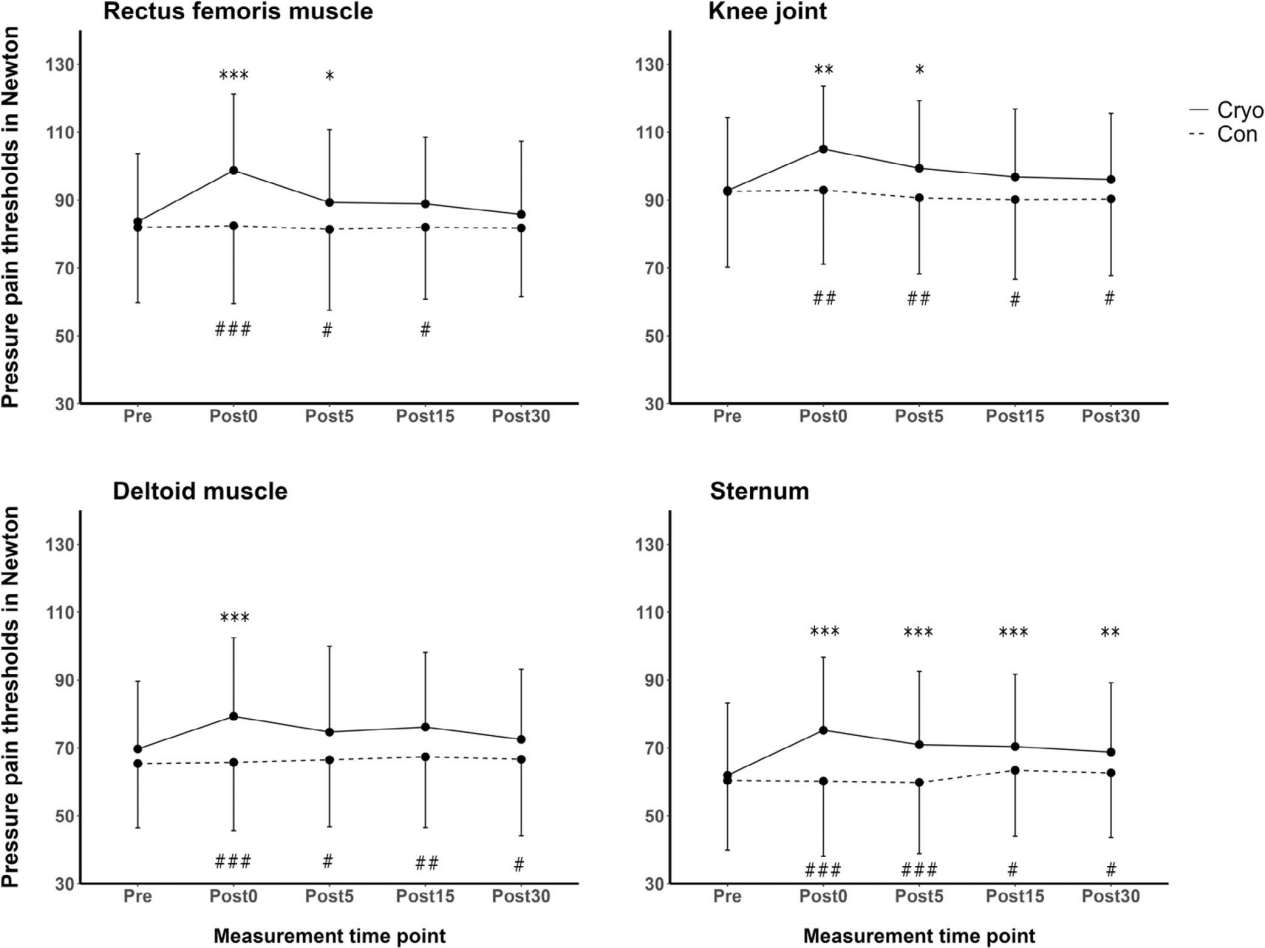
Changes in pressure pain thresholds separated by landmarks of participants (*n* = 24) before and after the cryo chamber intervention (Cryo) and control condition (Con). *p ≤ 0.05, **p ≤ 0.01, ***p ≤ 0.001 for within‐group differences compared to Pre. ^#^ *p* ≤ 0.05, ^##^ *p* ≤ 0.01, ^###^ *p* ≤ 0.001 for between‐group differences at the respective time point. Data are presented as mean with whiskers representing standard deviation.

Results of the three‐way ANOVA yielded significant ‘time’ × ‘condition’ interaction effects (*p* < 0.001, *η* ^2^_partial_ = 0.293). Pairwise post hoc testing for this interaction effect revealed higher Post0 (*p* < 0.001), Post5 (*p* = 0.001), Post15 (*p* = 0.002) and higher post30 (*p* = 0.025) values following the cryo chamber intervention compared to Pre. No such differences were observed for the control condition for any post time point (all *p* > 0.567). Besides, significant differences were observed between both interventions for Post0 (*p* < 0.001), Post5 (*p* < 0.001), Post15 (*p* = 0.001) and Post30 (*p* = 0.002), but not for Pre (*p* = 0.318). The respective post hoc test results of landmark‐specific PPT are presented in Figure 4. Additionally, results showed no significant interaction effect for ‘time’ × ‘condition’ × ‘landmark’ (*p* = 0.439, *η* ^2^_partial_ = 0.041), ‘landmark’ × ‘condition’ (*p* = 0.397, *η* ^2^_partial_ = 0.042) and ‘time’ × ‘landmark’ (*p* = 0.248, *η* ^2^_partial_ = 0.052). Descriptive data of raw data and log10‐transformed data of PPT are presented in Tables S1 and S2, respectively.

**FIGURE 5:**
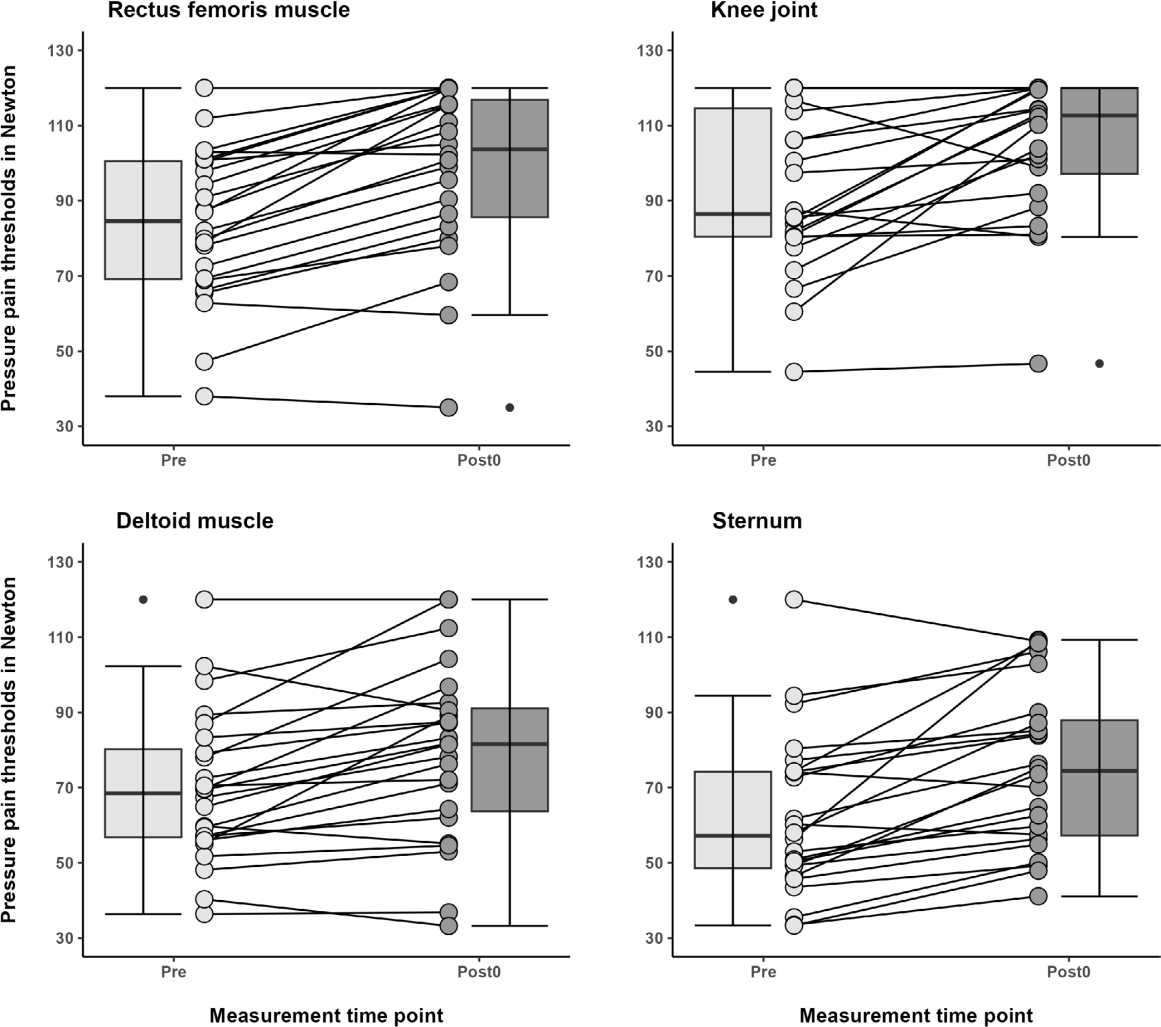
Average and individual response of pressure pain thresholds before (Pre) and immediately after (Post0) the cryochamber intervention in participants (*n* = 24). Data are presented as boxplots: Horizontal bars reflect the median, the box reflects the first and third quartile, and whiskers indicate 1.5 times the interquartile range. Outliers are displayed separately.

The immediate response to the cryochamber intervention showed that 82.6% of participants experienced an increase in PPT values (exceeding the SEM) between the Post0 and Pre assessments for PPT_Total_. At the RF, KJ, DM and ST, the proportion of immediate responders amounted to 82.6%, 72.2%, 56.5% and 87.0%, respectively (Figure 5). Additional data, including the number of responders clustered by landmarks and across all time points, are presented in Table S3.

## 4 Discussion

The primary aim of this study was to explore whether a 3‐min exposure to a cryochamber cooled to −87°C results in cryo‐induced hypoalgesia in healthy individuals. Besides, it was evaluated how long these results last and whether differences between PPT outcomes measured at bony and muscular landmarks exist. The results indicate a significant increase in PPT in general and at specific muscular and bony landmarks immediately after the cryochamber intervention compared to the control condition. Hence, cryo‐induced hypoalgesia was observed in this study. A significant elevation in PPT relative to baseline values was observed for up to 30 min post intervention for PPT_Total_, though with a gradual decline over time. However, the duration of the cryo‐induced hypoalgesic effect varied between landmarks, with the longest‐lasting effect detected at the ST, up to 30 min after leaving the cryochamber. Effects of the cryochamber intervention on pain perception appeared to not be dependent on the landmark assessed as no significant interaction effect was found. Moreover, the observed hypoalgesia for PPT_Total_ was found to be robust, with 82.6% of participants being classified as responders.

The analgesic potential of cryogenic environments has been studied repeatedly in various patient populations, yet pain‐related outcomes are mostly limited to pain scales (Adie et al. 2012; Garcia et al. 2021; Gizińska et al. 2015; Silva et al. 2022). However, little is known about the effect of cryogenic temperatures on experimental pain metrics such as pain sensitivity. Algafly and George investigated the impact of a locally applied ice pack to the ankle joint on pain sensitivity and pain tolerance. The authors concluded that when skin temperatures were reduced to 15°C and 10°C, pain sensitivity decreased with pain thresholds being significantly elevated (Algafly and George 2007). This observation was explained by the authors by the cooling‐induced reduction in afferent nerve conduction velocity (NCV). They suggested that NCV decreases progressively with reductions in skin temperature, with an average NCV reduction of 33% from baseline to 10°C equating to a 0.4 m/s decrease per 1°C drop in skin temperature (Algafly and George 2007). In the present study, this slowing of signal transmission of primary afferent nerve fibres most likely also happened due to the observed reductions in skin temperature (see Table 2). This might have artificially elevated PPT due to delayed nociceptive input processing. If the signal transmission from the peripheral stimulus site to the brain were slowed on a biophysical level, the actual mechanical pressure applied at the time of perception may have exceeded the true pain threshold. This effect would occur independently of a genuine hypoalgesic response, instead representing a distinct mechanism that contributes to the observed hypoalgesia. However, future studies are needed to evaluate NCV or alternative pain assessment methods to clearly differentiate potential methodological influences from true analgesic effects of cryotherapy.

In a separate study by Vargas E Silva et al., pain sensitivity and pain tolerance thresholds were measured following unilateral application of a thermal gel pack to the right knee joint for 20 min, with the contralateral site serving as an unmanipulated control. Despite uniformly increased thresholds at all landmarks assessed, statistically significant elevations in PPT were only observed at two out of the six measurement sites, despite significant temperature reductions at all landmarks (Vargas E Silva et al. 2019). In the present study, for the first time, PPT were measured after application of a three‐minute whole‐body cryotherapy within a cryochamber cooled to −87°C. Whole‐body cryotherapy differs from the above‐mentioned localised cryotherapy applications by providing systemic rather than localised cooling. Whole‐body cryotherapy involves exposing the entire body to extremely low standardised temperatures resulting in a rapid reduction in skin surface temperature, and it triggers a cascade of not only localised but also central (e.g., central nervous system related) physiological responses (Cholewka et al. 2006; Louis et al. 2020). It is suggested that whole‐body cryotherapy induces a complex endocrine response characterised by an increase of certain neurotransmitters, along with adaptations on the metabolic and hormonal level (Nasi et al. 2020). Although not directly measured in the present study, acute endocrine responses, particularly of cortisol, catecholamines (adrenaline, noradrenaline), and β‐endorphin, may further explain the observed PPT response in the present study from a physiological perspective (Barłowska‐Trybulec et al. 2022; Leppäluoto et al. 2008; Pertovaara 2013). Despite comparable skin temperature reductions of local and whole‐body cryo applications on a local level, it can be assumed that subjecting the entire body to cryogenic temperatures elicits a higher magnitude of transmitter release, resulting in more profound mechanical hyperalgesia. However, both application modalities are known to induce an upregulation of the autonomic nervous system, explaining in part their analgesic effects (Bouzigon et al. 2016). Evidence comparing localised to whole‐body cryotherapy modalities is scarce and needs to be elaborated on in further studies.

The results presented herein are concordant with the study by Algafly and George (Algafly and George 2007), with PPT being significantly increased in general and at all landmarks immediately post intervention, although absolute local temperatures were on average comparably higher at Post0 (lowest at RF: 20.4; highest at ST: 27.8). The biggest and smallest relative reduction in temperature between Pre and Post were observed at the RF (35.4%) and ST (17.9%), respectively. Interestingly, according to a previous review, skin temperature needs to be lowered to 13.6°C for local analgesia (Bleakley and Hopkins 2010). The authors state that reaching skin temperatures that low is difficult, which was mirrored by temperature values achieved after the cryochamber in this study. As our results suggest significant increases in PPT across all landmarks assessed at higher absolute skin temperatures, current recommendations do not apply when pain perception is assessed by means of PPT. Although, it needs to be highlighted that within this investigation only healthy individuals were tested.

It is well established and logically sensible that superficial body temperatures decrease when subjected to ice packs directly applied to the body site assessed or when the body remains in a cryogenic environment for some time. This phenomenon has been widely studied and recognised (Cuttell et al. 2017; Hausswirth et al. 2013; Louis et al. 2020). However, this effect lasts only for a brief time period after withdrawal from the cryogenic environment. The results of the present study substantiate these findings. Superficial temperatures were significantly reduced at Post0 compared to baseline values for all landmarks examined. However, temperature exponentially increased after 5, 15 and 30 min, respectively. Physiological processes are initiated as a consequence of extreme cooling of the body to account for the homeostatic control of body temperature regulated mainly by neurons residing in the hypothalamus (Zhao et al. 2017).

Analogous to changes in temperature, exposure to cryogenic temperatures induces an immediate positive chronotropic effect on the heart. HR increased by 40.7% upon entering the cryochamber from baseline measured during the control condition and remained significantly elevated immediately after withdrawal from the cryochamber (+22.4% compared to baseline), albeit with an incipient decrease. These findings are in concordance with a recent study by Coppi and colleagues (Coppi et al. 2022) that explored the response of the cardiovascular system to 2‐min whole‐body cryotherapy, in which a 10.6% increase in HR was observed in non‐professional athletes.

### 4.1 Strengths and Limitations and Future Directions

The main strengths of this study are that the pain physiological phenomenon of cryo‐induced hypoalgesia was evaluated in a randomised and controlled crossover design by the use of semi‐objective PPT. Besides, the use of a cryochamber with a predefined temperature of −87°C ensures highest standardisation of applied temperatures, which can be seen as superior to other cryotherapy methods or localised applications. However, some limitations need to be acknowledged. First, due to the nature of the cryo‐application, blinding of the participants and tester was not possible. Second, the effects of the cryo‐application were measured up to 30‐min post. Future research should aim to also explore more delayed (e.g., 24‐h post) effects of cryogenic application methods on pain perception. Third, this study aimed to explore a physiological phenomenon and only healthy male participants were included, thus limiting the generalisability of the results observed. Future research should aim to reproduce this study in, for instance, pain patients. Fourth, this study explored the acute effects of a cryo‐application on pain perception and no assumptions on chronic effects (e.g., resulting from repetitive multi‐week exposures) can be drawn from this work. Future research should therefore investigate whether repeated exposures to cryogenic temperatures within a cryochamber also result in habituation in terms of experimental pain perception. Fifth, experimental pain stimuli are limited to mechanical pressure applications and no conclusions can be drawn regarding other stimulus types (e.g., thermal or chemical). Lastly, this study was not prospectively registered.

## 5 Conclusion

This study for the first time demonstrated that a 3‐min exposure to a cryochamber cooled to −87°C results in general robust cryo‐induced hypoalgesia with increased PPT compared to a control condition. This hypoalgesia was observed immediately after the intervention and persisted for up to 30 min, though it gradually declined over time. Importantly, no significant differences in the response were found between different landmarks, suggesting that the cryochamber intervention had a consistent impact on pain perception across measurement sites. These results suggest that whole‐body cryotherapy represents an effective, non‐invasive method for providing short‐term pain relief that should be replicated in patients with chronic or acute pain conditions.

## Supporting information


Data S1.

## Data Availability

The data that support the findings of this study are available from the corresponding author upon reasonable request.
