# Supplementary material for: Cryo‐Induced Hypoalgesia: The Effects of an Acute Cryochamber Exposure on Pain Perception—A Randomised Controlled Cross‐Over Trial
Source: Eur J Pain. 2025 Apr 4;29(5):e70017. doi: 10.1002/ejp.70017 (PMC11971597; doi:10.1002/ejp.70017)
Supplement: Supplementary file 1 — Data S1. [file EJP-29-0-s001.docx]

**Supplementary Table 1.** Raw data of pressure pain thresholds across all measurement time points (n=24)

| Landmark | Condition | Pre | Post0 | Post5 | Post15 | Post30 |
| --- | --- | --- | --- | --- | --- | --- |
| PPT_Total_ | Cryo  Con | 77.0 ± 17.2  (39.1-120.0)  75.1 ± 18.8  (38.0-116.8) | 89.6 ± 18.6  (39.0-117.3)  75.3 ± 19.4  (40.5-118.3) | 83.6 ± 19.4  (41.7-118.0)  74.6 ± 19.  (36.4-116.2) | 83.1 ± 18.2  (47.5-118.5)  75.7 ± 19.3  (36.9-112.9) | 80.8 ± 17.7  (51.4-118.9)  75.3 ± 19.1  (41.2-116.2) |
| Rectus  femoris | Cryo  Con | 83.7 ± 20.1  (38.0-120.0)  81.9 ± 22.1  (32.7-120.0) | 98.7 ± 22.5  (35.0-120.0)  82.4 ± 23.0  (41.4-120.0) | 89.3 ± 21.4  (49.6-120.0)  81.4 ± 23.9  (41.0-120.0) | 88.9 ± 19.6  (50.5-120.0)  82.0 ± 21.1  (41.8-120.0) | 85.8 ± 21.6  (55.2-120.0)  81.8 ± 20.1  (45.6-120.0) |
| Deltoideus | Cryo  Con | 69.7 ± 20.0  (36.4-120.0)  65.4 ± 18.9  (30.1-110.5) | 79.3 ± 23.2  (33.2-120.0)  65.7 ± 20.2  (29.2-119.7) | 74.7 ± 25.3  (28.1-120.0)  66.5 ± 19.7  (30.0-120.0) | 76.2 ± 22.0  (30.6-120.0)  67.4 ± 20.8  (24.8-114.2) | 72.5 ± 20.6  (29.1-119.7)  66.7 ± 22.5  (30.1-120.0) |
| Knee joint | Cryo  Con | 92.8 ± 21.5  (44.5-120.0)  92.5 ± 22.3  (48.4-140.0) | 105.1 ± 18.5  (46.7-120.0)  93.0 ± 21.8  (53.7-120.0) | 99.4 ± 19.9  (46.6-120.0)  90.7 ± 22.5  (40.0-120.0) | 96.8 ± 20.0  (50.0-120.0)  90.1 ± 23.4  (44.6-120.0) | 96.1 ± 19.5  (63.5-120.0)  90.3 ± 22.6  (48.0-120.0) |
| Sternum | Cryo  Con | 62.0 ± 21.2  (33.3-120.0)  60.4 ± 20.6  (35.4-116.6) | 75.2 ± 21.4  (41.1-109.3)  60.2 ± 22.1  (31.2-113.3) | 71.0 ± 21.6  (36.1-116.9)  59.8 ± 21.0  (23.3-104.7) | 70.4 ± 21.3  (39.7-114.7)  63.4 ± 19.4  (26.7-99.7) | 68.8 ± 20.4  (37.3-116.0)  62.6 ± 19.0  (29.2-104.8) |

Data presented as mean ± standard deviation (Min-Max). Con = control condition, Cryo = cryochamber condition.

**Supplementary Table 2.** Log10-transformed data of pressure pain thresholds across all measurement time points (n=24)

| Landmark | Condition | Pre | Post0 | Post5 | Post15 | Post30 |
| --- | --- | --- | --- | --- | --- | --- |
| PPT_Total_ | Cryo  Con | 1.876 ± 0.102  (1.590-2.080)  1.861 ± 0.115  (1.580-2.070) | 1.941 ± 0.105***  (1.590-2.070)  1.862 ± 0.118^###^  (1.610-2.070) | 1.910 ± 0.110***  (1.620-2.070)  1.858 ± 0.121^###^  (1.560-2.070) | 1.908 ± 0.102**  (1.680-2.070)  1.864 ± 0.123^###^  (1.570-2.050) | 1.897 ± 0.099*  (1.710-2.080)  1.863 ± 0.117^###^  (1.610-2.070) |
| Rectus  femoris | Cryo  Con | 1.909 ± 0.117  (1.544-2.079)  1.895 ± 0.135  (1.515-2.079) | 1.980 ± 0.126***  (1.544-2.079)  1.900 ± 0.129^###^  (1.617-2.079) | 1.938 ± 0.113*  (1.695-2.079)  1.891 ± 0.137^#^  (1.613-2.079) | 1.938 ± 0.103  (1.703-2.079)  1.899 ± 0.119^#^ (1.621-2.079) | 1.920 ± 0.113  (1.742-2.079)  1.900 ± 0.109  (1.659-2.079) |
| Deltoideus | Cryo  Con | 1.826 ± 0.124  (1.561-2.079)  1.797 ± 0.136  (1.479-2.043) | 1.879 ± 0.144***  (1.521-2.079)  1.798 ± 0.137^###^  (1.465-2.078) | 1.845 ± 0.167  (1.449-2.079)  1.804 ± 0.135^#^  (1.477-2.079) | 1.862 ± 0.138  (1.486-2.079)  1.805 ± 0.155^##^  (1.394-2.058) | 1.841 ± 0.138  (1.464-2.078)  1.798 ± 0.156^#^  (1.479-2.079) |
| Knee joint | Cryo  Con | 1.955 ± 0.110  (1.648-2.079)  1.953 ± 0.114  (1.685-2.079) | 2.013 ± 0.094**  (1.669-2.079)  1.955 ± 0.111^##^  (1.730-2.079) | 1.987 ± 0.099*  (1.668-2.079)  1.943 ± 0.119^##^  (1.602-2.079) | 1.976 ± 0.098  (1.699-2.079)  1.939 ± 0.126^#^  (1.649-2.079) | 1.974 ± 0.092  (1.803-2.079)  1.941 ± 0.118^#^  (1.681-2.079) |
| Sternum | Cryo  Con | 1.769 ± 0.144  (1.522-2.079)  1.758 ± 0.143  (1.549-2.067) | 1.859 ± 0.127***  (1.614-2.039)  1.753 ± 0.155^###^  (1.494-2.054) | 1.831 ± 0.135***  (1.558-2.068)  1.750 ± 0.161^###^  (1.367-2.020) | 1.828 ± 0.136***  (1.599-2.060)  1.780 ± 0.149^#^  (1.427-1.999) | 1.819 ± 0.128**  (1.572-2.064)  1.776 ± 0.140^#^ (1.465-2.020) |

Data presented as mean ± standard deviation (Min-Max). Con = control condition, Cryo = cryochamber condition,
*p ≤ 0.05, **p ≤ 0.01, ***p ≤ 0.001 for within-group differences compared to Pre-values.
^#^p ≤ 0.05, ^##^p ≤ 0.01, ^###^p ≤ 0.001 for between-group differences compared to the cryochamber condition.

**Supplementary Table 3.** Number of responders of the cryochamber intervention in participants (n=24)

| Landmark | # Responder  Post0-Pre | # Responder  Post5-Pre | # Responder  Post15-Pre | # Responder  Post30-Pre |
| --- | --- | --- | --- | --- |
| PPT_Total_ | 19/23  (82.6%) | 12/23  (52.2%) | 12/23  (52.2%) | 7/16  (30.4%) |
| Rectus femoris | 19/23  (82.6%) | 13/23  (56.5%) | 11/23  (47.8%) | 9/24  (39.1%) |
| Knee joint | 13/18  (72.2%) | 10/18  (55.6%) | 7/18  (38.9%) | 11/18  (61.1%) |
| Deltoid muscle | 13/23  (56.5%) | 9/23  (39.1%) | 13/23  (56.5%) | 11/23  (47.8%) |
| Sternum | 20/23  (87.0%) | 15/23  (65.2%) | 13/23  (56.5%) | 14/23  (60.9%) |

Only subjects with baseline value of PPT smaller than 120 Newton - respective SEM were considered.
Responder: difference between post measurement and pre > SEM of baseline PPT.
